# All Is Not Loss: Plant Biodiversity in the Anthropocene

**DOI:** 10.1371/journal.pone.0030535

**Published:** 2012-01-17

**Authors:** Erle C. Ellis, Erica C. Antill, Holger Kreft

**Affiliations:** 1 Department of Geography & Environmental Systems, University of Maryland, Baltimore, Maryland, United States of America; 2 Biodiversity, Macroecology & Conservation Biogeography Group, Georg-August University of Göttingen, Göttingen, Germany; Umea University, Sweden

## Abstract

Anthropogenic global changes in biodiversity are generally portrayed in terms of massive native species losses or invasions caused by recent human disturbance. Yet these biodiversity changes and others caused directly by human populations and their use of land tend to co-occur as long-term biodiversity change processes in the Anthropocene. Here we explore contemporary anthropogenic global patterns in vascular plant species richness at regional landscape scales by combining spatially explicit models and estimates for native species loss together with gains in exotics caused by species invasions and the introduction of agricultural domesticates and ornamental exotic plants. The patterns thus derived confirm that while native losses are likely significant across at least half of Earth's ice-free land, model predictions indicate that plant species richness has increased overall in most regional landscapes, mostly because species invasions tend to exceed native losses. While global observing systems and models that integrate anthropogenic species loss, introduction and invasion at regional landscape scales remain at an early stage of development, integrating predictions from existing models within a single assessment confirms their vast global extent and significance while revealing novel patterns and their potential drivers. Effective global stewardship of plant biodiversity in the Anthropocene will require integrated frameworks for observing, modeling and forecasting the different forms of anthropogenic biodiversity change processes at regional landscape scales, towards conserving biodiversity within the novel plant communities created and sustained by human systems.

## Introduction

Human populations and their use of land have transformed more than three quarters of the terrestrial biosphere into anthropogenic biomes (anthromes; [1]), both by replacing native ecosystems with agriculture and settlements and by managing and disturbing the remnant and recovering ecosystems embedded within these used lands [2]–[4]. This direct anthropogenic transformation of the terrestrial biosphere is causing unprecedented global changes in biodiversity as native species are driven to extinction locally and globally [5]–[12] and domestic and exotic species are rapidly becoming established [13]–[17].

Native global patterns of plant species richness have long been known to follow global patterns of latitude, climate, and topography [18]–[20]. However, anthropogenic global patterns of plant species richness remain poorly understood, despite their undoubted importance to ecology and conservation, in part because human activities simultaneously cause native species losses and exotic species gains [7], [14]–[16], [21], [22] and in part because anthropogenic changes in biodiversity tend to be viewed as recent disturbances that can and must be contained, reduced, or eliminated (e.g. [6], [8], [9], [12]).

In the Anthropocene, anthropogenic changes in biodiversity are neither temporary nor fully avoidable: they are the inevitable, predictable and potentially manageable consequences of sustained human residence and use of land together with the interactive effects of global climate change [2], [4], [7], [22], [23]. This study presents the first spatially explicit integrated assessment of the anthropogenic global patterns of vascular plant species richness created by the sustained actions of human populations and their use of land at regional landscape scale [24]. To accomplish this, a set of basic global models and estimates of anthropogenic species gains and losses were used to predict contemporary global patterns of plant species richness within regional landscapes, which we define here by stratifying Earth's ice-free land surface into equal-area hexagonal grid cells of 7800 km^2^, a spatial scale well within the size range of the regional landscape units generally used to characterize regional and subregional patterns in biodiversity at the global scale [24]. We then use these modeled and estimated richness data to explore what these can tell us about the global patterns of plant species richness created by human populations and their use of land across biomes, anthromes, biogeographic realms, and biodiversity hotspots.

### A simple integrated model of anthropogenic species richness (*ASR*)

Anthropogenic species richness (*ASR*) results when humans interact with native patterns of species richness. Within a given area, *ASR* can be quantified as the sum of native species richness (*N*), anthropogenic loss of native species (*ASL*) and anthropogenic species increase (*ASI*):
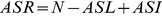
(1)


*ASL* within a given area is commonly predicted as a function of *N* and the area of native habitat lost to agriculture and settlements (*HL*) by applying classic species-area relationships (SAR; [25]). *ASI* within a given area may be estimated as the sum of exotic species invasions (*IS*), agricultural domesticates (crop species; *CS*), and exotic ornamental species (*OS*) present in an area. Estimating *ASR* based on these very basic assumptions oversimplifies and even omits some key processes by which humans alter plant species richness, including global climate change [23], exotic displacement of natives [16], [26], and interactions among these and other processes [6], [23]. Nevertheless, these assumptions offer a practical starting point for model-based estimates of *ASR*, *ASL,* and *ASI* that summarize the state of current knowledge of anthropogenic global patterns of plant species richness and may serve as hypotheses against which more comprehensive data and models may be tested in the future. While these estimates must therefore be considered preliminary, we present these with the aim of stimulating global change and biodiversity science as well as the conservation community to embrace a more comprehensive and long-term view of the novel anthropogenic patterns of biodiversity sustained by human systems in the Anthropocene [2], [4], [27].

## Results and Discussion

The use of global models and empirical estimation procedures (Appendix S1) enabled quantitative assessment of a wide variety of anthropogenic changes in plant species richness patterns across the terrestrial biosphere. Most estimates incorporated substantial uncertainties, as indicated by upper and lower error bounds presented in square brackets. Detailed global estimates are provided in Appendix S2; supplemental maps and links to downloadable data are in Appendix S3.

### The big picture: all is change

**Figure 1:**
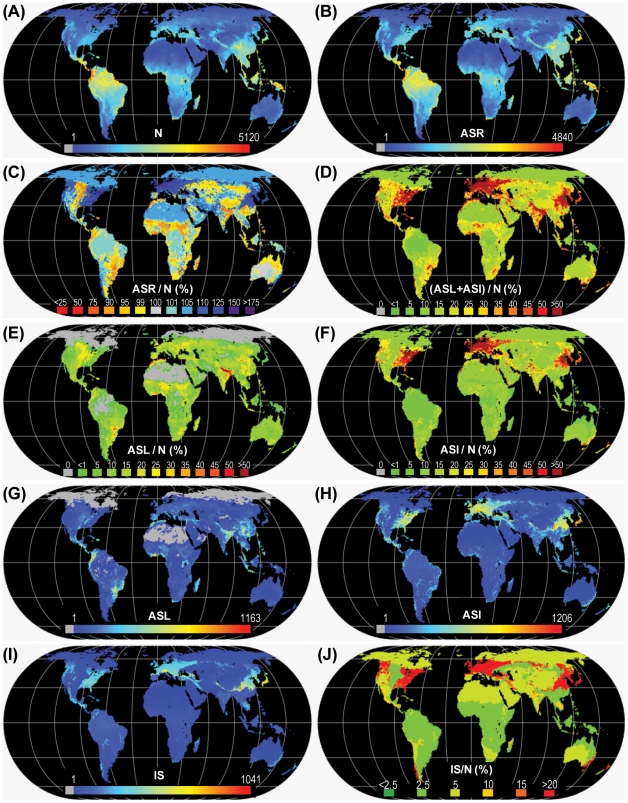
Global maps of (A) Native species richness (*N*), (B) Anthropogenic species richness (*ASR*), (C) Anthropogenic species richness (*ASR*) relative to *N*, (D) total anthropogenic species loss (*ASL*) + anthropogenic species increase (*ASI*) relative to *N*, (E) *ASL* relative to *N*, (F) *ASI* relative to *N*, (G) *ASL*, (H) *ASI*, (I) exotic species invasions (*IS*), (J) *IS* relative to *N*. All maps in Eckert IV global equal area projection.

Global model predictions indicate that human populations and their use of land have substantially altered plant species richness within regional landscapes across most of the terrestrial biosphere (93% [35–100%] of ice-free land area), either by causing at least 5% of their native species to be lost, or by introducing exotic species at levels equivalent to 5% or more of *N*, or both (Figure 1). Model results indicate that at least 5% of native species appear to have been lost from regional landscapes in half of the terrestrial biosphere (51% [14–64%]), and more than a quarter of the biosphere may have lost more than 10% of its natives, resulting in a median loss of 5% [1.4–14%] of *N* across regional landscapes globally (Figures 1E, 1G). Yet this widespread local loss of native species is not the greatest change predicted by global models.

Most regional landscapes (89% [31–100%] of ice-free land) have likely experienced substantial increases in exotic species, by ≥5% of *N*, with exotics exceeding 10% of native richness across more than a quarter of the biosphere, causing a median global exotic increase of 7% [3–18%] relative to *N* (Figures 1F, 1H). Anthropogenic species gains exceeded losses across more than two thirds of the terrestrial biosphere (69% [37–100%]) and exceeded losses by more than 5% of *N* across almost half of the terrestrial biosphere (47% [6–99%]; Figure 1C). While these results are striking, they agree well with previous studies indicating that human-induced losses of native species from regional landscapes are usually much lower than anthropogenic increases resulting from species invasions [15], [21], [28]–[30]. Indeed, modeled ratios of species invasions to total native species were nearly identical (*IS*/*N* ∼20%) to those observed by Stohlgren et al. [21] in forest-dominated states of the US Pacific Northwest; one of the most heavily invaded regions globally (Figure 1J; [14]), and also agree in general with estimates across a variety of regions by Vitousek et al. [13] and across European Nations by Chytrý et al. [31], [32](if both neophytes and archaeophytes are considered). However, exotic invasions appeared to be substantially underestimated (by 2/3) in US states dominated by grasslands and deserts when compared with Stohlgren et al. [21]. More importantly, our SAR model predicted far higher levels of species loss than observed in all states, by a factor of 3 to 16 [21]. On average, species invasions accounted for the vast majority of exotic species introductions in most regional landscapes (79% [67–87%]), with crops averaging 13% and ornamentals just 8% of exotic species introductions. This was mostly because ornamentals, while abundant where present, are cultivated primarily in urban and urbanizing regional landscapes which cover only about 14% of global land in this assessment (Appendices S1 and S2).

### Changing by staying the same

**Figure 2:**
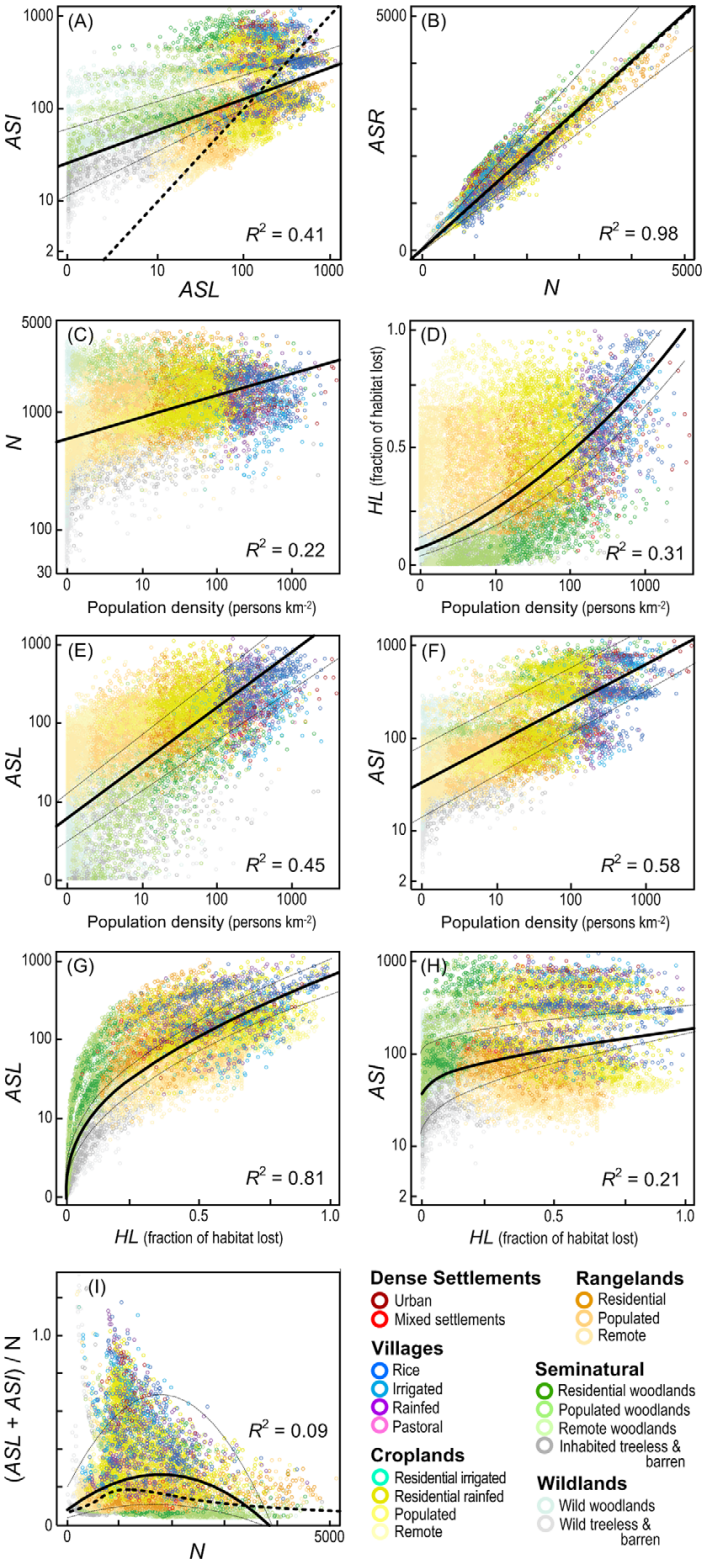
Global relationships between (A) anthropogenic species loss (*ASL*) and increase (*ASI*), (B) anthropogenic (*ASR*) and native (*N*) species richness, (C) *N* and population density, (D) Habitat loss (*HL*; fraction of habitat lost to land use) and population density, (E) *ASL* and population density and (F) *ASI* and population density, (G) *ASL* and *HL*, and (H) *ASI* and *HL*. Points represent regional landscape cells, colored by anthrome class. Thick black lines are regressions with *R* ^2^ at lower right; thin dashed black lines are upper and lower regression models from sensitivity analysis. Thick dashed black lines indicate *X* = *Y* in (A) and (B) and smoothed curve fit to data in (I).

Though it appears that the vast majority of the terrestrial biosphere has gained or lost a substantial number of species (Figure 1D), models indicate that only about half of the biosphere (61% [22–100%]) has experienced a substantial net anthropogenic change in plant species richness (by 5% of *N* or more; Figure 1C). For this reason, anthropogenic global patterns of plant species richness (*ASR*; Figure 1A) still strongly resemble native patterns (*N*; Figure 1B), and nearly 98% of global variation in *ASR* can be predicted by global variation in *N* (Figure 2B). Indeed, in describing global variations in *ASR*, *N* had many times the predictive power (0.92) of exotic species invasions (*IS*: 0.13; Figures 1I, 1J), species loss (*ASL*: 0.09; Figures 1E, 1G), ornamentals (*OS*: 0.07), or crop introductions (*CS*: 0.03), based on standardized coefficients from multiple regression (*P*<0.001 model; all variables *R* ^2^>0.99).

The apparent persistence of native global patterns of plant species richness in the face of widespread gains and losses has many possible explanations, all relating to the balancing of species losses with species gains. A simple explanation is that our models, by predicting both species loss and exotic species invasions partly as a function of *N*, have created artificial relationships between loss and gain, balancing out their relative effects (*R* ^2^ for *N* vs. *ASL* = 0.44, *N* vs. *ASI* = 0.54; *N* vs. *IS* = 0.57). Yet an even simpler explanation is random chance. Substantial gains occurred across 89% [31–100%] of the terrestrial biosphere and substantial losses across 51% [14–64%] (Figures 1E, 1F, 1G, 1H), so gains would be expected to offset losses at random across roughly 45% of the biosphere (45% = 89%×51%); a value very similar to the 39% [0–78%] of the biosphere without a substantial net anthropogenic change in species richness (*ASR*/*N* between 95% and 105%; Figure 1C). Nevertheless, the positive relationship between anthropogenic species increase and loss was significantly stronger than chance (*AS*I *vs. ASL R* ^2^ = 0.41; Figure 2A) and may represent, at least by proxy, a basic global pattern by which humans alter biodiversity.

### Anthropogenic succession: thinning globally, enriching locally

The basic global pattern by which humans appear to have altered plant species richness in regional landscapes is by causing moderate loss of native species (Figure 1E) coupled with related but larger gains in exotic species (Figures 1F, 2A), mostly by invasions (Figures 1I, 1J; [15], [21]). Traditionally, this coupling of species gain and loss has been explained by the equilibrium concept of community saturation [26], in which ecological succession maintains relatively constant “saturated” levels of species richness under a given set of environmental conditions, thereby sustaining the classic biogeographic patterns of species richness [18]–[20]. By this theory, when humans and other disturbances cause native species loss, “vacant” niches are formed, and these may then be filled back to native richness levels by exotics [33]–[35]. Alternatively, species invasions may themselves cause disturbance and native loss, or exotics may simply displace natives from their ecological niches directly by competition [33], [35]. In any case, richness levels are constrained mainly by the abiotic environment, and net anthropogenic gains result only from temporary disequilibrium conditions brought about by human disturbance; predisturbance richness would presumably return were equilibrium restored by the elimination of human disturbance [35]. Yet, evidence against community saturation is accumulating, in part from observations on invaded communities [21], [26], [28].

A general theory of anthropogenic ecological succession may help explain the mounting evidence that exotic species gains appear to correlate with and exceed native losses (Figure 2A). Simply put, the same anthropogenic drivers that cause native species losses may facilitate exotic species gains in similar but greater measure. As human populations establish, grow more dense, and develop, they first use land extensively and later intensify their use in the most optimal environments, releasing marginal lands to regenerate as novel ecosystems, all the while becoming better connected materially with other human systems [4], [36], [37]. While land use for agriculture and settlements reduces native habitat area, land-use intensification follows rising populations, and these also drive an ever-accelerating flow of propagules along human trade and transport networks, facilitating exotic introductions and their establishment in the remnant and recovering novel habitats embedded within used and settled landscapes [2], [17], [37]. The result would be what we find today: increasingly globalized and homogenized anthropogenic plant communities characterized by reduced native richness but enriched in species at the regional landscape scale by exotics drawn globally from the relatively small pool of species that either tolerate or benefit from the novel anthropogenic habitats created by human residence and use of land [2], [33].

Assessing land use and population as global drivers of anthropogenic ecological succession is a challenge because of their complex interrelationships. Land use drives habitat loss and varies with human population density (Figure 2D; [1]) and all three correlate with native species richness (Figure 2C; *HL* vs. *N R* ^2^ = 0.12; [29], [32], [38], [39]. Yet the relative strengths of their global relationships are revealing. Human population density is a remarkably strong predictor of both anthropogenic species loss and gain (Figures 2E, 2F) even though it was not used in any of our models and was only weakly linked to *N* and *HL*, which were used (Figures 2C, 2D). Habitat loss was a surprisingly weak predictor of anthropogenic species increase (*HL* vs. *ASI*; Figure 2H), given that habitat loss is directly related to land use and therefore strongly coupled with both crop and ornamental species richness. Population density was also a better predictor than habitat loss of overall changes in species richness ((*ASI*+*ASL*)/*N*; *R* ^2^ = 0.47 vs. 0.32; data not shown). Taken together, these results indicate that human population density, which drives land use intensification, might ultimately be an even better indicator of anthropogenic ecological change than land use or habitat loss [1], [32].

### Where the wild things are (and aren't)

Whatever the mechanism, by enriching plant communities with exotic species and thinning native species locally and globally, humans are causing a vast biotic homogenization of plant communities across the terrestrial biosphere [17], [30], [33], [40], [41]. Based on existing models and estimates, the net result is a terrestrial biosphere in which almost half of regional landscapes are enriched substantially by exotic plant species when compared with undisturbed native richness (Figures 1C, 2B). And while an additional 39% [0–78%] of the biosphere seems without a substantial net change in species richness, this was only because exotic gains offset native losses.

**Figure 3:**
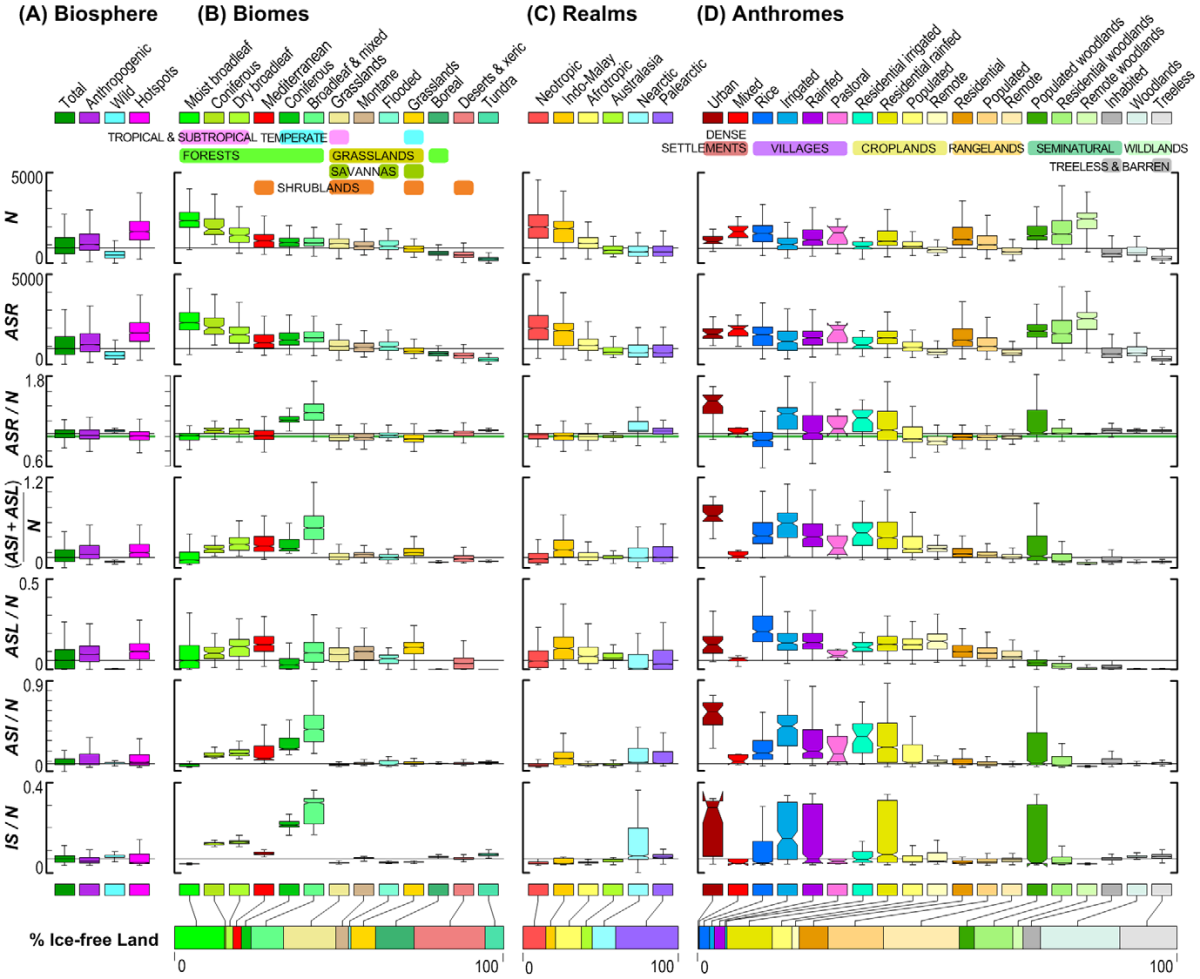
Global patterns of plant species richness and its changes across (A) the terrestrial biosphere, (B) biomes (C) biogeographic realms and (D) anthromes. Notch in box plots is 95% confidence interval for median; whiskers exclude outliers. Horizontal black lines are global medians; green line in *ASR*/*N* plot highlights *ASR* = *N*. Horizontal bar charts at bottom present class areas in proportion to their global area.

Today, few native plant communities remain undisturbed and without exotic companions (Figure 1D). Though wild areas have retained their native species, they also appear to be comparatively rich in exotics (Figures 1E, 3A). Only 31% [0–63%] of regional landscapes had less plant species after anthropogenic alteration (*ASR*≤*N*; Figure 1C) and only in 14% [0–53%] of regional landscapes were net declines in species richness substantial (≥5% of *N*; Figure 1C). Net declines were present mostly in regions where native losses were highest (Figures 1E) and therefore exceeded gains, especially in the grasslands, savannas, shrublands (50% of total unenriched area) and deserts (16%) of Northern Eurasia, Central North America, Sub-Saharan and Southern Africa and Australia, and in the moist tropical forests of the Neotropics and Madagascar (Figure 3B) in regions dominated by rangelands (65%) and croplands (20%; Figure 3D).

As observed globally, anthropogenic species richness usually followed native richnesspatterns across biomes and biogeographic realms (Figures 3B, 3C). Temperate forests were the main exceptions, with large net increases in species richness, primarily by invasions [14]. Ornamentals were also especially abundant in the temperate broadleaf and mixed forest biomes, comprising about one third of all introduced exotics, helping to explain why this biome had the highest anthropogenic species enrichment observed across biomes (*ASR*/*N ∼*132%; Figure 3B) and all but the urban anthrome (147%; Figure 3D). While most biomes lost natives, the Mediterranean biome lost the most natives (median *ASL*/*N* = 14%; Figure 3B), followed by tropical and subtropical dry broadleaf forests and temperate grasslands. Temperate grasslands also had the largest overall net biome-level declines in species richness (*ASR*/N; Figure 3B), primarily because these had the highest levels of habitat loss across biomes (median *HL* = 66%; Appendix S2).

Biodiversity hotspots ([42]; 32 of 34 present in this study) generally followed trends observed across the biosphere as a whole, especially those for the anthropogenic biosphere (Figure 3A; individual hotspot statistics in Appendix S2). This despite the fact that hotspots tended to be more intensively used (median habitat loss, *HL* = 40%) and densely populated (median population = 29 persons km^−2^) than the terrestrial biosphere as a whole (28% *HL*, 3.4 persons km^−2^) and much of the anthropogenic biosphere (42% *HL*, 11 persons km^−2^). Nevertheless, eight hotspots appeared to have lost more than 10% of native species from their regional landscapes - substantially more than the median for the anthropogenic biosphere (median = 8%). While no hotspot gained more non-natives than the temperate broadleaf forest biome (41% of *N*), six hotspots gained more than 20% non-natives. As with the rest of the biosphere, anthropogenic species increase usually balanced native species loss, and only four hotspots showed a substantial net decline in species richness. Exceptionally large non-native gains were observed in two hotspots (Japan 39%, California 30%) as a result of extremely high levels of invasion in Japan, and high invasions plus high ornamentals in California. In terms of total species gains and losses, the most anthropogenically altered hotspot overall was Japan (median = 42%), and the wildest was the East Melanesian Islands, with species loss near zero and the lowest anthropogenic species increase relative to native levels as well (*ASI*/*N ∼* 4%; note that our models do not include island effects, which are considerable). Still, 18 of 32 hotspots had a greater total species loss + gain than the global median for the anthropogenic biosphere (18%), confirming that the most biodiverse regions on Earth also tend to be among the most challenged by anthropogenic transformation.

### The anthropogenic melting pots

Patterns of plant species richness across anthromes reveal the strong global coupling of human and natural systems (Figure 3D; [38], [39]). In keeping with global trends, native species richness and anthropogenic species richness, loss, and increase all tended to increase with human population density in anthromes (Figure 3D; “Residential”, “Populated” and “Remote” define populations of 10 to 100, 1 to 10 and >0 to 1 per km^2^, respectively [1]). The only anthromes with substantial net declines in species richness were remote croplands and rice villages, and these also had the highest median habitat loss (*HL* 73% and 65%) and native species losses (16 and 21% respectively) observed across anthromes, biomes, realms and hotspots (Figure 3D; Appendix S2).

Unsurprisingly, the highest levels of net species increase (*ASR*/*N*) and overall human alteration ((*ASI*+*ASL*)/*N*; Figure 3D) were found in the most densely populated and most intensively used of anthromes (urban, village and residential croplands), in part because of their exceptional abundance of ornamentals (35% to 62% of *ASI*; Appendix S2). The least used anthromes, the seminatural woodlands, were also the most species rich, even more so than wild woodlands (Figure 3D), as these were predominantly Tropical and Subtropical while wild woodlands are now mostly Boreal. Overall, these trends confirm that humans appear to have preferentially settled in, used, and most profoundly altered temperate regions, which have intermediate levels of plant species richness (*N ∼*1000 species/cell), while leaving the most species rich and species poor regions less intensively used for agriculture and less densely populated (Figures 2I, 3D; [4], [29], [36]).

### Biogeography for an anthropogenic biosphere

After more than a century of scientific effort, what we don't know about the global patterns of plant species richness still exceeds what we do know, and this is probably true of most other organisms except possibly land mammals and birds [5], [6]. The plant species richness patterns we have presented here, though based on the strongest empirical models and estimates presently available for regional landscapes at global scales, remain hypothetical. Moreover, the biodiversity changes caused directly by human populations and their use of land may ultimately be considered minor if anthropogenic climate change continues unabated [11], [23], [43].

Even native patterns of plant species richness remain poorly documented for many taxa in many geographic regions and must be inferred from statistical models [19], [20]. The global distributions of major crop species are increasingly well documented [44] but these represent only a fraction of domesticated plants, and the global diaspora of ornamental species is especially understudied. Considering the species richness of botanical gardens, some cities might sustain as many as 10^4^ exotic species, many potentially invasive [45]. It is therefore likely that the ornamental species richness estimates used in this assessment are overly conservative, potentially underestimating exotic richness in densely populated regions by an order of magnitude or more (Appendix S1).

While species invasions are widely studied on a case by case and regional basis, they are not well understood globally, especially in forests outside of the temperate zone [14], [30], [37], [46], [47]. Perhaps because of this, global patterns of plant species invasion have yet to be linked empirically with anthropogenic drivers like transportation networks or economics even though such links almost certainly exist [14], [17], [29], [47]–[51]. Global patterns of native species loss from regional landscapes might appear to be well understood because of their theoretical coupling with the loss of native habitat, yet model predictions based on this theory tend to perform poorly for a variety of reasons [5], [11], [52]. The theoretical model predictions of this study were no exception, and appear to greatly overestimate losses when compared with observations at regional landscape scale [21]. While this might be the result of “extinction debt” [53], documented cases of plant species extinctions remain far smaller than expected based on classic habitat models and remain a great challenge to observe or predict [11], [52]; the plant species losses we estimate for regional landscapes can shed little light on global extinctions. Given that generational time is required to observe extinction processes [54], sustained monitoring of native populations in anthropogenic landscapes will be necessary, especially to ensure that the longer lived species of vascular plants are reproducing adequately; many of these may already be living fossils- or emerging domesticates- if artificial propagation is required to avert extinctions.

As massive species invasions tend to correlate with and overwhelm native species losses, neither of these alone are now adequate as general indicators of anthropogenic changes in biodiversity [6], [7], [21], [22], [53]. Indicators that combine native species loss and exotic species gain and relate these to native richness may prove more robust as general indicators of human influence on biodiversity (Figures 1D, 2I, 3), though their precise ecological meanings have yet to be explored [22]. And species richness is only a beginning. In the end, species diversity, evenness, and the functional and phylogenetic diversity of communities are most important to understanding biodiversity and its role in ecosystem function, and these are not necessarily linked tightly to species richness [6], [22], [55], [56].

However biodiversity is measured, progress in understanding its global patterns and their anthropogenic changes is held back by the absence of systematic and standardized global observations at regional landscape scales [7], [24], [57]–[59]. To make these observations useful for understanding, forecasting and conserving plant diversity across the terrestrial biosphere in the Anthropocene, these must integrate native species losses and exotic species gains and couple them with spatially explicit models that include data on human population densities, land use, transportation networks, economics and other direct anthropogenic drivers of ecological succession, together with the classic abiotic drivers of diversity [18] and anthropogenic changes in these [7], [23], [27], [47], [60].

### All is not loss: sustaining biodiversity in anthromes

Human reshaping of ecological pattern and process is global, profound, and in most cases virtually irreversible, making it more than a challenge to conserve most species in native habitats. With rare exceptions, it is already too late to keep human influence away from Earth's biodiversity hotspots or anywhere else. Yet all is not lost. Despite widespread losses of native species and even greater increases in exotics caused by invasions, domesticates and other intentional introductions, anthropogenic patterns of plant species richness still appear to strongly resemble native patterns across the terrestrial biosphere. Even in ancient agricultural villages (Figure 3D) and urban domestic gardens [61], the most densely populated and intensively-used anthromes, the majority of native plant species appear to be sustaining viable populations, though in the shadow of their more abundant exotic competitors – a pattern of change in plant species assemblages resembling those observed during prior mass extinctions in the fossil record (which are based on losses of Marine taxa; [62]). Moreover, given the apparent linkage of human population densities with both native loss and exotic species gain (Figures 2E, 2F), rural population declines caused by rapid urbanization may already be causing native species recoveries in developing regions [4], [32].

It may still be possible to sustain most of Earth's plant species within the exotic-enriched anthromes that now make up most of the terrestrial biosphere, especially if anthropogenic ecological succession can be redirected to sustain native plant species as part of multifunctional land management strategies that incorporate biodiversity as a valued benefit together with agriculture and other land uses [17], [27], [63]–[66]. Accomplishing this will require fundamental advances in global scientific understanding of how native species can be conserved within the novel plant communities created and sustained by human systems across most of the terrestrial biosphere in the Anthropocene [2], [4], [27], [56], [66], [67].

## Methods

### Characterizing *ASR* globally

Global patterns in vascular plant species richness within regional landscapes were assessed by first dividing Earth's ice-free land surface into 16,805 hexagonal cells, each with a total area of approximately 7,800 km^2^ (95 km between cell centers; Appendix S1).

Native and anthropogenic species richness, loss and increase within regional landscape cells were estimated using theoretical models and estimates as outlined below and detailed in Appendix S1. *N* was estimated using the species richness model of Kreft & Jetz [20] (Figure 1A), rescaled to fit the area of the regional landscape cells of this assessment. *ASL* was estimated from *N* within each cell using biome-level empirical vascular plant SAR models [19] and native habitat areas remaining in each cell estimated after subtracting agriculture and urban settlements (*HL*; calculated from Klein Goldewijk et al. [68], [69]. Crop species (*CS*) were estimated from Monfreda et al. [44], and ornamental domesticates (*OS*) from urban area and published counts of urban exotic domestic plant species (Appendix S1). Exotic species invasions were estimated using Lonsdale's [14] empirical models relating species invasions to *N* within broadly-defined biomes. Finally, *ASI* was calculated as the sum of *CS*, *OS* and *IS*, and *ASR* was calculated by equation 1.

The significance of anthropogenic changes in plant species richness was assessed relative to native conditions by dividing *ASI*, *ASL* and other richness estimates by *N* within each cell; changes greater than 5% of *N* will be termed “substantial” here, though changes far less than this may also represent profound alterations of biodiversity and ecosystem function. Global relationships between species richness, gains and losses were explored using regression analysis after appropriate transformation (log_10_+1 for species numbers and population density, square root for *HL*). Uncertainties in model predictions for *ASL*, *IS*, *OS* and the estimates derived from them (*ASI*, *ASR*) were characterized using upper and lower error bounds derived from a worst case sensitivity analysis (Appendix S1) and included in square brackets where appropriate.

## Supporting Information

Appendix S1 Methods and data used for global analysis.
(PDF)

Appendix S2 Global statistics
(XLS)

Appendix S3 Maps and spatial data.
(PDF)
