# Correction: All is not loss: Plant biodiversity in the anthropocene

**DOI:** 10.1371/journal.pone.0327912

**Published:** 2025-07-09

**Authors:** Erle C. Ellis, Erica C. Antill, Holger Kreft

There is an error in affiliation 1 for authors Erle C. Ellis and Erica C. Antill. The correct affiliation 1 is: Department of Geography & Environmental Systems, University of Maryland, Baltimore County, Baltimore, Maryland, United States of America.
