# Supplementary material for: All Is Not Loss: Plant Biodiversity in the Anthropocene
Source: PLoS One. 2012 Jan 17;7(1):e30535. doi: 10.1371/journal.pone.0030535 (PMC3260302; doi:10.1371/journal.pone.0030535)
Supplement: Appendix S1 — Methods and data used for global analysis. (PDF) [file pone.0030535.s001.pdf]

Ellis E.C., Antill, E.C. and H. Kreft (in review):
All is Not Loss: Plant Biodiversity in the Anthropocene
PLOSone

APPENDIX S1: Methods and data used for global analysis.

Spatial data structure. Earth's ice-free land area was stratified for analysis into a global set of equal area hexagonal grid cells using a geodesic discrete global grid system (DGG) with a median cell area of 7792 km^2 (mean +/- SD = 7791.4 +/- 9.6 km^2; Level 8 DGG), with an intercell spacing of approximately 95 km, defined on an icosahedron and projected to the sphere using the inverse Icosahedral Snyder Equal Area (ISEA) Projection (Sahr 2003). The DGG was projected to an Eckert IV Equal area projection using a Geographic Information System (GIS) to calculate cell areas. These DGG cells are an order of magnitude smaller than most global ecoregions (e.g. Olson et al. 2001; median ecoregion area = 56,300 km^2) and were used as standard global units for regional landscape estimates (Noss 1990).

Global data inputs. Global data for land area, human population density, and percent cover by urban, crop, and pasture lands at 5 arc minute resolution were obtained from the HYDE 3.1 data model (Klein Goldewijk et al. 2010a,b) and used to estimate areas and populations within each DGG cell using zonal mean statistics calculated using GIS. Cells with <50% cover by land were removed, as were islands of one or two cells, leaving a total of 16805 cells with a global land area of 125,018,446 km^2 (97.5% of original land area = 128,185,612 km^2). Habitat loss (*HL*) was estimated as the sum of land area occupied by urban settlements and crops and two thirds of pasture area, under the assumption that pasture lands were incompletely cultivated and less intensively used than croplands or urban areas and therefore retain substantial native habitat. Biomes and biogeographic realm maps (Olson et al. 2001) and biodiversity hotspot maps (Mittermeier et al. 2005) in vector GIS format were intersected with DGG cells and classified based on the class with largest area within each cell. Anthrome maps (v2; Ellis et al. 2010) were mapped to the DGG based on the zonal majority anthrome class determined within each cell.

Native species (*N*) model. Current knowledge of the global distribution of native vascular plant species richness (*N*) remains fragmentary, though both observations and models are improving (e.g. Barthlott et al., 1996; Barthlott et al., 2005; Kier et al., 2005; Kleidon and Mooney, 2000; Kreft and Jetz, 2007; Kreft et al., 2008, Woodward & Kelly, 2008). Ideally, global patterns in species richness would be mapped by quantifying overlaps in the geographic ranges of individual species (e.g. Buckley and Jetz, 2007; Grenyer et al., 2006; Orme et al., 2005; Stuart et al., 2004). However, this approach is not yet feasible for vascular plants because species' ranges are insufficiently documented in most regions of the world (Kreft & Jetz, 2007; Yesson et al., 2007). Here, the number of native vascular plant species (*N*) per DGG cell was estimated using the species richness model of Kreft & Jetz (2007). This model is based on empirical statistical relationships between environment (potential evapotranspiration, number of wet days per year, topographic and habitat heterogeneity, and three-dimensional vegetation complexity) and species richness patterns obtained from datasets documenting the number of native species in 1032 geographic regions worldwide. Additionally, the floristic kingdoms are included as dummy variables to control for regional effects, potentially reflecting idiosyncratic, historical factors contributing to broad-scale richness gradients. Predicted species numbers per cell ranged from 5 to 5,120 per c. 7,800 km^2 (mean = 1,086, median = 842, standard deviation = 812), a range of values consistent with other global assessments of plant species numbers at similar spatial scales (Barthlott et al., 2005; Kier et al., 2005).

Anthropogenic species loss (*ASL*) model. We used the well-established power model of the species-area relationship (SAR; Arrhenius, 1921; Rosenzweig, 1995) to predict losses of native species within each grid cell caused by loss of native habit (*ASL* = anthropogenic species loss). SAR power models estimate the number of species (*S*) present in a given area (*A*) as: $S=c*A^z$, based on empirical constants *c* and *z*. For log-log transformed data, the constant *c* represents the intercept and *z* the slope of the SAR. This relationship is widely used to predict species extinctions caused by habitat loss (e.g. Brooks et al., 1997; Simberloff, 1984; Thomas et al., 2004).

The number of native species present after a given area of habitat is lost (S_{new}) can be predicted using a simple transformation: $S_{new}/S_{ori} = (A_{new}/A_{ori})^z$, where S_{ori} is the original number of native species, A_{ori} the original area, and A_{new}, the reduced area after habitat loss. The challenge of this approach is to determine *z*, the SAR slope or the rate at which species become extirpated as habitat area is reduced. Previous studies (e.g. Thomas et al., 2004) have used a global slope with constant value close to 0.25 (Drakare et al., 2006; Preston, 1962), though this simplification has been criticized (Harte et al., 2004) and SAR slopes are known to vary geographically along gradients of latitude or climate (Kier et al., 2005; Qian et al., 2007; Williams et al., 2009). There is also a time lag between habitat loss and observed local or regional extinction (Brook et al. 2003, Turner et al. 1994), an “extinction debt” (Tilman et al., 1994) that may be especially pronounced in long-lived plant species.

Two options for using SAR to estimate global patterns in *ASL* within regional landscapes were considered. First, as with previous studies, a globally constant SAR slope of *z* = 0.25 might be applied to estimates of habitat loss by conversion to crops, pastures and urban settlements. Alternatively, we might apply separate, empirically derived SAR slopes for vascular plants derived for 13 biomes worldwide by Kier et al. (2005: Table 1). These values vary between 0.11 for deserts and xeric shrublands and 0.33 for the Tropical and subtropical moist broadleaf forest biome in Central America (Kier et al., 2005: Table 1) and are on average significantly smaller than the common SAR slope of 0.25. To obtain more conservative (lower) and more spatially-sensitive *ASL* estimates, we therefore used Kier et al.’s (2005) biome-level SAR slopes rather than a constant global SAR slope of 0.25.

Invasive species (*IS*) model. After more than five decades of studying biological invasions (Elton 1958), global-scale patterns of invasive species richness remain elusive. Despite increased efforts in invasion observation and tracking, we were unable to locate a global dataset of invasive species that is both spatially explicit and empirical. There are many good reasons for this, including the limited spatial extent of observations and challenges in determining whether a given species is invasive and when invaders have become naturalized. At present, available data on *IS* numbers are extremely biased geographically towards North temperate regions (e.g. Lonsdale 1999, Qian et al. 2006, Stohlgren et al. 2003; Winter et al. 2009) severely hampering global modeling.

Biological invasions are an outcome of a complex interaction between anthropogenic influences (e.g. number of introduced propagules, geographic patterns of human population size, trade, and infrastructure) as well as ecological and biogeographical effects (e.g. regional invasibility, climatic constraints, biotic interaction). At broad-geographic scales and for mainland regions ecologists frequently reported strong positive correlations between *N* and *IS* (Lonsdale 1999; Stohlgren et al. 2003). The precise mechanism is still debated, but this pervasive trend allows a rough approximation of *IS* using *N*.

Lonsdale (1999: Table 3, Model 4) reports a global statistical relationship between *N* and *IS*: $\log IS = -0.27 + 0.82 * \log N$. This model was derived from 177 floras worldwide. Applying this global relationship overestimates *IS* in some biomes (e.g. wet tropics, deserts) while it underestimates *IS* especially in temperate agricultural and urban regions because it produces an average proportion of invasive species of about 19%. Lonsdale further reports that the average proportions of *IS* worldwide vary tremendously between 6 and 31 %.
This suggests a significant interaction between biome membership and *N* in predicting *IS*. We thus adjusted the intercepts according to the average proportions of invasive plants in Lonsdale's eight coarse biomes reported in Lonsdale's Table 6 (Table 1). Therefore, all grid cells grid cells were assigned to one of Lonsdale's biomes (compare Table). This yielded more realistic average proportions of *IS* in different biomes. Nevertheless, due to the limited data underpinning most of these global relationships these *IS* estimates should be considered very rough and preliminary.

Table 1: Mean proportions of invasive species (*IS/(IS+N)*) present in different biomes as reported by Lonsdale (1999; Table 6) and linked to Olson biomes (Table 2) with adjusted intercepts.

**class**	**Lonsdale biomes***	**Olson biome class**	**mean *IS* / (*IS+N*)** (%)	**adjusted intercept**
1	Temperate agricultural/urban	4, 5 and only cells with ≥100 persons km^{-1} or crop cover ≥ 20%	31	0.06
2	Temperate forest	4,5 and only cells not included in class 1 (above)	22	-0.14
3	Tropical & subtropical dry or coniferous forests†	2, 3	7	-0.29
4	Mediterranean shrubland	12	17	-0.51
5	Alpine	6, 10, 11	11	-0.66
6	Savanna	7, 8, 9	8	-0.8
7	Wet tropics	1	6	-0.8
8	Desert	13	6	-0.72

* Lonsdale's (1999) "multiple" biome class was defined at continental scales and therefore too large for this analysis.
† Class not present in Lonsdale (1999); *IS/(IS+N)* approximated as mean of classes 6 and 8.

Table 2: Olson Biome Classes (Olson et al. 2001)

**class**	**label**
1	Tropical & subtropical moist broadleaf forests
2	Tropical & subtropical dry broadleaf forests
3	Tropical & subtropical coniferous forests
4	Temperate broadleaf & mixed forests
5	Temperate coniferous forests
6	Boreal forests
7	Tropical & subtropical grasslands, savannas & shrublands
8	Temperate grasslands, savannas & shrublands
9	Flooded grasslands & savannas
10	Montane grasslands & shrublands
11	Tundra
12	Mediterranean forests, woodlands & shrublands

13	Deserts & xeric shrublands
14*	Mangrove
98*	Lakes
99*	Barren- snow and ice

*not used

Crop Species (*CS*) data. The total number of crop species (*CS*) were estimated within each DGG cell by counting all crop species with area >0 in a 5 arc minute gridded dataset quantifying the cultivated areas of 175 crop species areas prepared by Monfreda et al. (2008), with a maximum of 79 crop species observed in any cell. This *CS* analysis certainly undercounts crop species in any given cell both because many crop species are missing from the dataset to begin with, or are aggregated into categories of crop species. Further, only areas of national and commercial agricultural significance are usually reported to Food and Agriculture Organization of the United Nations, the base data source for this dataset (Monfreda et al. 2008), leaving out the less common crop species grown in smaller amounts by smallholders and consumed locally. As a rough estimate, we approximate that the current *CS* estimates undercount by at least 30% the crop species actually present in any given cell. Further, these errors are not evenly distributed, as much greater numbers of missing crop species would be expected in the most densely populated agricultural regions and in the tropics.

Ornamental species (*OS*) estimates. We define ornamental plant species (*OS*) as non-native plants introduced intentionally for non-agricultural purposes and maintained in association with human settlements and other built structures, including yards, gardens, parks, roadsides, and other infrastructure. Based on the limited literature available, *OS* are highly abundant in urban areas, but global relationships between *OS* and other variables, including *N*, have yet to be observed, with the exception of city age, for which there currently exist no consistent global data (La Sorte et al. 2007). We therefore estimated *OS* based on the extent of urban areas within each DGG cell based on a pooled median estimate of 439 non-native plant species richness observed across a sample of 20 cities in the USA and Europe (range = 186 to 823; La Sorte et al. 2007, Loram et al. 2009) and 3 “domestic gardens” (range = 523 to 798; in southeastern France, Mexico City, and Sheffield, UK; Marco et al. 2008). DGG cells with substantial urban areas (>5% cover, >390 km^2 per cell) were assigned an OS value of 400 species, cells with significant urban areas (between 0.5% and 5% cover) were assigned an OS value of 200 species, and cells with <0.5% urban land cover were set to zero. While we were initially concerned that some species might be double-counted as both *OS* and *IS*, existing studies indicate that only about 1% of introduced species become established outside cultivation, based on the so-called "tens rule": 1/10 of introductions are found in the wild (10%), 1/10 of these become established (1%) and 1/10 of these become pests (0.1%) Williamson and Fitter 1996, Richardson and Pysek 2006, Smith et al. 2006). Based on the fact that DGG cells are much larger than cities (~7800 km^2 vs. 1,707 km^2 for London, UK), there is little doubt that our *OS* estimates are very conservative, likely underestimating actual *OS* by at least 50% or more. Finally, we do not consider botanic gardens, which would significantly increase *ASR*, potentially by many thousands of species (Golding et al. 2010), but have a very limited spatial extent and small population sizes.

Worst Case Sensitivity Analysis. Uncertainties in model predictions for *ASL*, *IS*, *OS* and the estimates derived from them (*ASI*, *ASR*) were characterized by upper and lower error bounds derived from a worst case sensitivity analysis (Morgan and Henrion 1990: p. 178). Errors in estimating *N* (Kreft and Jetz 2007) and land use areas (Klein Goldewijk et al. 2010a,b) used in calculating *HL* are ignored for this analysis. Conservative upper and lower bounds for errors

in model predictions were obtained by making model predictions using extreme upper and lower values for model inputs derived either from model data sources, or when uncertainties were unknown, by computing lower input values as ½ and upper input values as 2 times the standard value of model inputs.

Errors in *ASL* were estimated using upper and lower estimates for two model inputs: *z*, the slope of SAR model, and the proportion of pasture land conversion that is considered "lost habitat". Error estimates for *z* values were not provided by Kier et al. (2005), so these were estimated as ½ and 2 times the values in Kier et al.'s (2005) Table 1). Standard estimates of habitat loss by land conversion to pasture was set to 2/3 of pasture area; lower and upper estimates of this parameter were set to 1/3 and 100% of pasture area. Lower error bounds for *ASL* estimates were then made by simultaneously using the lower *z* and lower pasture habitat loss values when computing *ASL*; upper error bounds were estimated using the upper values.

Errors in the linear *IS* model parameters were computed using error estimates provided by Lonsdale (1999). The empirical slope used in the model (0.82) was increased or decreased by two standard errors (SE; 2 x 0.07), and coupled with empirical intercepts for each Lonsdale biome (Table 1) increased or decreased by two SE computed from the standard deviations and sample sizes provided in Lonsdale's Table 6 (the Wet Tropics biome had just one observation; SE for this biome was set to 1.5 times the maximum SE across biomes). Lower and upper *OS* estimates were produced simply by halving and doubling their standard estimates.

Once upper and lower error bounds for model outputs were estimated for all regional landscape cells, all derived estimates were calculated, including *ASI* and *ASR*. Upper error limits for *ASR* were obtained by combining high *ASI* with low *ASL* and lower errors from low *ASI* and high *ASL*.

REFERENCES

Arrhenius, O. 1921. Species and area. Journal of Ecology 9:95-99.

Barthlott, W., W. Lauer, and A. Placke. 1996. Global distribution of species diversity in vascular plants: towards a world map of phytodiversity. Erdkunde 50:317-328.

Barthlott, W., J. Mutke, M. D. Rafiqpoor, G. Kier, and H. Kreft. 2005. Global centres of vascular plant diversity. Nova Acta Leopoldina 92:61-83.

Brook, B. W., N. S. Sodhi, and P. K. L. Ng. 2003. Catastrophic extinctions follow deforestation in Singapore. Nature 424:420-423.

Brooks, T. M., S. L. Pimm, and N. J. Collar. 1997. Deforestation predicts the number of threatened birds in insular Southeast Asia. Conservation Biology 11:382-394.

Buckley, L. B. and W. Jetz. 2007. Environmental and historical constraints on global patterns of amphibian richness. Proceedings of the Royal Society of London Series B-Biological Sciences 274:1167-1173.

Drakare, S., J. J. Lennon, and H. Hillebrand. 2006. The imprint of the geographical, evolutionary and ecological context on species-area relationships. Ecology Letters 9:215-227.

Ellis, E. C., K. Klein Goldewijk, S. Siebert, D. Lightman, and N. Ramankutty. 2010. Anthropogenic transformation of the biomes, 1700 to 2000. Global Ecology and Biogeography 19:589-606.

Golding, J., Güsewell, S., Kreft, H., Kuzevanov, V.Y., Lehvävirta, S., Parmentier, I. & Pautasso, M. 2010. Species-richness patterns of the living collections of the world's botanic gardens: a matter of socio-economics? Annals of Botany 105: 689-696

Grenyer, R., C. D. L. Orme, S. F. Jackson, G. H. Thomas, R. G. Davies, T. J. Davies, K. E. Jones, V. A. Olson, R. S. Ridgely, P. C. Rasmussen, T.-S. Ding, P. M. Bennett, T. M. Blackburn, K. J. Gaston, J. L. Gittleman, and I. P. F. Owens. 2006. Global distribution and conservation of rare and threatened vertebrates. Nature 444:93-96.

Kier, G., J. Mutke, E. Dinerstein, T. H. Ricketts, W. Küper, H. Kreft, and W. Barthlott. 2005. Global patterns of plant diversity and floristic knowledge. Journal of Biogeography 32:1107-1116.

Kleidon, A. and H. A. Mooney. 2000. A global distribution of biodiversity inferred from climatic constraints: results from a process-based modelling study. Global Change Biology 6:507-523.

Klein Goldewijk, K., A. Beusen, and P. Janssen. 2010a. Long-term dynamic modeling of global population and built-up area in a spatially explicit way: HYDE 3.1. The Holocene 20:565-573.

Klein Goldewijk, K., A. Beusen, G. van Drecht, and M. de Vos. 2010b. The HYDE 3.1 spatially explicit database of human induced global land use change over the past 12,000 years. Global Ecology & Biogeography: in press.

Kreft, H. and W. Jetz. 2007. Global patterns and determinants of vascular plant diversity. Proceedings of the National Academy of Sciences of the United States of America 104:5925-5930.

Kreft, H., W. Jetz, J. Mutke, G. Kier, and W. Barthlott. 2008. Global diversity of island floras from a macroecological perspective. Ecology Letters 11:116-127.

La Sorte, F. A., M. L. McKinney, and P. Pysek. 2007. Compositional similarity among urban floras within and across continents: biogeographical consequences of human-mediated biotic interchange. Global Change Biology 13:913-921.

Lonsdale, W. M. 1999. Global patterns of plant invasions and the concept of invasibility. Ecology 80:1522-1536.

Loram, A., K. Thompson, P. H. Warren, and K. J. Gaston. 2009. Urban domestic gardens (XII): The richness and composition of the flora in five UK cities. Journal of Vegetation Science 19:321-330.

Marco, A., T. Dutoit, M. Deschamps-Cottin, J.-F. Mauffrey, M. Vennetier, and V. Bertaudière-Montes. 2008. Gardens in urbanizing rural areas reveal an unexpected floral diversity related to housing density. Comptes Rendus Biologies 331:452-465.

Mittermeier, R. A., P. R. Gil, M. Hoffmann, J. Pilgrin, T. Brooks, C. G. Mittermeier, J. Lamoreux, and G. A. B. Fonseca. 2005. Hotspots revisited. Earth's biologically richest and most endangered terrestrial ecoregions. CEMEX, New Mexico.

Monfreda, C., N. Ramankutty, and J. A. Foley. 2008. Farming the planet: 2. Geographic distribution of crop areas, yields, physiological types, and net primary production in the year 2000. Global Biogeochemical Cycles 22:GB1022.

Morgan, M. G. and M. Henrion. 1990. Uncertainty: a Guide to Dealing with Uncertainty in Quantitative Risk and Policy Analysis. Cambridge University Press, New York, New York, USA.

Noss, R. F. 1990. Indicators for monitoring biodiversity: a hierarchical approach. Conservation Biology 4:355-364.

Olson, D. M., E. Dinerstein, E. D. Wikramanayake, N. D. Burgess, G. V. N. Powell, E. C. Underwood, J. A. D'Amico, I. Itoua, H. E. Strand, J. C. Morrison, C. J. Loucks, T. F. Allnutt, T. H. Ricketts, Y. Kura, J. F. Lamoreux, W. W. Wettengel, P. Hedao, and K. R. Kassem. 2001. Terrestrial ecoregions of the world: a new map of life on Earth. BioScience 51:933-938.

Orme, C. D. L., R. G. Davies, M. Burgess, F. Eigenbrod, N. Pickup, V. A. Olson, A. J. Webster, T.-S. Ding, P. C. Rasmussen, R. S. Ridgely, A. J. Stattersfield, P. M. Bennett, T. M. Blackburn, K. J. Gaston, and I. P. F. Owens. 2005. Global hotspots of species richness are not congruent with endemism or threat. Nature 436:1016-1019.

Preston, F. W. 1962. The canonical distribution of commonness and rarity. Ecology 43:185-215, 410-432.

Qian, H. and R. E. Ricklefs. 2006. The role of exotic species in homogenizing the North American flora. Ecology Letters 9:1293–1298.

Qian, H., J. D. Fridley, and M. W. Palmer. 2007. The latitudinal gradient of species-area relationships for vascular plants of North America. American Naturalist 170:690-701.

Richardson, D. M. and P. Pysek. 2006. Plant invasions: merging the concepts of species invasiveness and community invasibility. Progress in Physical Geography 30:409-431.

Rosenzweig, M. L. 1995. Species diversity in space and time. Cambridge University Press, Cambridge.

Sahr, K., D. White, and A. J. Kimerling. 2003. Geodesic discrete global grid systems. Cartography and Geographic Information Science 30:121(114).

Simberloff, D. S. 1984. Mass extinction and the destruction of moist tropical forests. Zhurnal Obshchei Biologii 45:767–778.

Smith, R. M., K. Thompson, J. G. Hodgson, P. H. Warren, and K. J. Gaston. 2006. Urban domestic gardens (IX): Composition and richness of the vascular plant flora, and implications for native biodiversity. Biological Conservation 129:312-322.

Stohlgren, T. J., D. T. Barnett, and J. T. Kartesz. 2003. The rich get richer: patterns of plant invasions in the United States. Frontiers in Ecology and the Environment 1:11-14.

Stuart, S. N., J. S. Chanson, N. A. Cox, B. E. Young, A. S. L. Rodrigues, D. L. Fischman, and R. W. Waller. 2004. Status and trends of amphibian declines and extinctions worldwide. Science 306:1783-1786.

Thomas, C. D., A. Cameron, R. E. Green, M. Bakkenes, L. J. Beaumont, Y. E. Collingham, B. F. N. Erasmus, M. Ferreira de Siqueira, A. Grainger, L. Hannah, L. Hughes, B. Huntley, A. S. van Jaarsveld, G. F. Midley, L. Miles, M. A. Ortega-Huerta, and e. al. 2004. Extinction risk from climate change. Nature 427:145-148.

Tilman, D., R. M. May, C. L. Lehman, and M. A. Nowak. 1994. Habitat destruction and the extinction debt. Nature 371:65-66.

Turner, I. M., H. T. W. Tan, Y. C. Wee, A. B. Ibrahim, P. T. Chew, and R. T. Corlett. 1994. A study of plant species extinction in Singapore: lessons for the conservation of tropical biodiversity. Conservation Biology 8:705-712.

Williams, M. R., B. B. Lamont, and J. D. Henstridge. 2009. Species-area functions revisited. Journal of Biogeography 36:1994-2004.

Williamson, M. and A. Fitter. 1996. The Varying Success of Invaders. Ecology 77:1661-1666.

Winter, M., O. Schweiger, S. Klotz, W. Nentwig, P. Andriopoulos, M. Arianoutsou, C. Basnou, P. Delipetrou, V. Didžiulis, and M. Hejda. 2009. Plant extinctions and introductions lead to phylogenetic and taxonomic homogenization of the European flora. Proceedings of the National Academy of Sciences 106:21721–21725.

Woodward, F. I. and C. K. Kelly. 2008. Responses of global plant diversity capacity to changes in carbon dioxide concentration and climate. Ecology Letters 11:1229-1237.

Yesson, C., P. W. Brewer, T. Sutton, N. Caithness, J. S. Pahwa, M. Burgess, W. A. Gray, R. J. White, A. C. Jones, and F. A. Bisby. 2007. How Global Is the Global Biodiversity Information Facility? PLoS One 2.
