# Supplementary material for: All Is Not Loss: Plant Biodiversity in the Anthropocene
Source: PLoS One. 2012 Jan 17;7(1):e30535. doi: 10.1371/journal.pone.0030535 (PMC3260302; doi:10.1371/journal.pone.0030535)
Supplement: Appendix S3 — Maps and spatial data. (PDF) [file pone.0030535.s003.pdf]

Ellis, E.C, Antill, E.C. and H. Kreft (in review):
All is Not Loss: Plant Biodiversity in the Anthropocene
PLOSone

Appendix S3: Maps and spatial data.

Spatial Data
For GIS analysis, in ESRI Shapefile format, downloadable at:
http://ecotope.org/anthromes/biodiversity/plants/data

Supplemental Maps (separate pages below)
All maps are Level 8 DGG grids (Appendix 1) in Eckert IV global equal area projection.
Figure 1: Biomes (Olson *et al.* 2001)
Figure 2: Biogeographic Realms (Olson *et al.* 2001)
Figure 3: Biodiversity Hotspots (Mittermeier *et al.* 2005)
Figure 4: Anthromes (v2; Ellis *et al.* 2010)
Figure 5: Crop Species Richness (*CS*; based on Monfreda *et al.* 2008; Appendix 1).
Figure 6: Ornamental Species Richness (*OS*; prepared as detailed in Appendix 1).

Figure 1. Biomes (Olson *et al.* 2001)

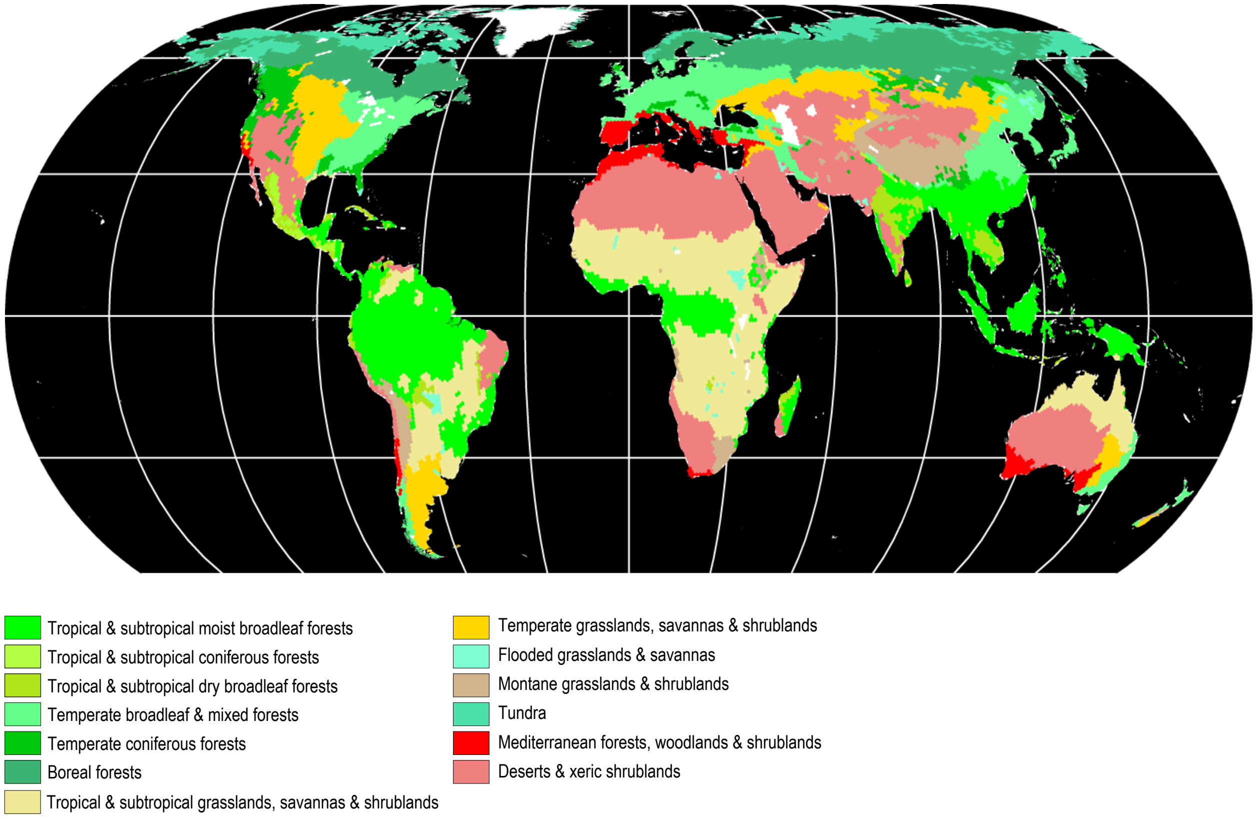

Figure 2: Biogeographic Realms (Olson *et al.* 2001)

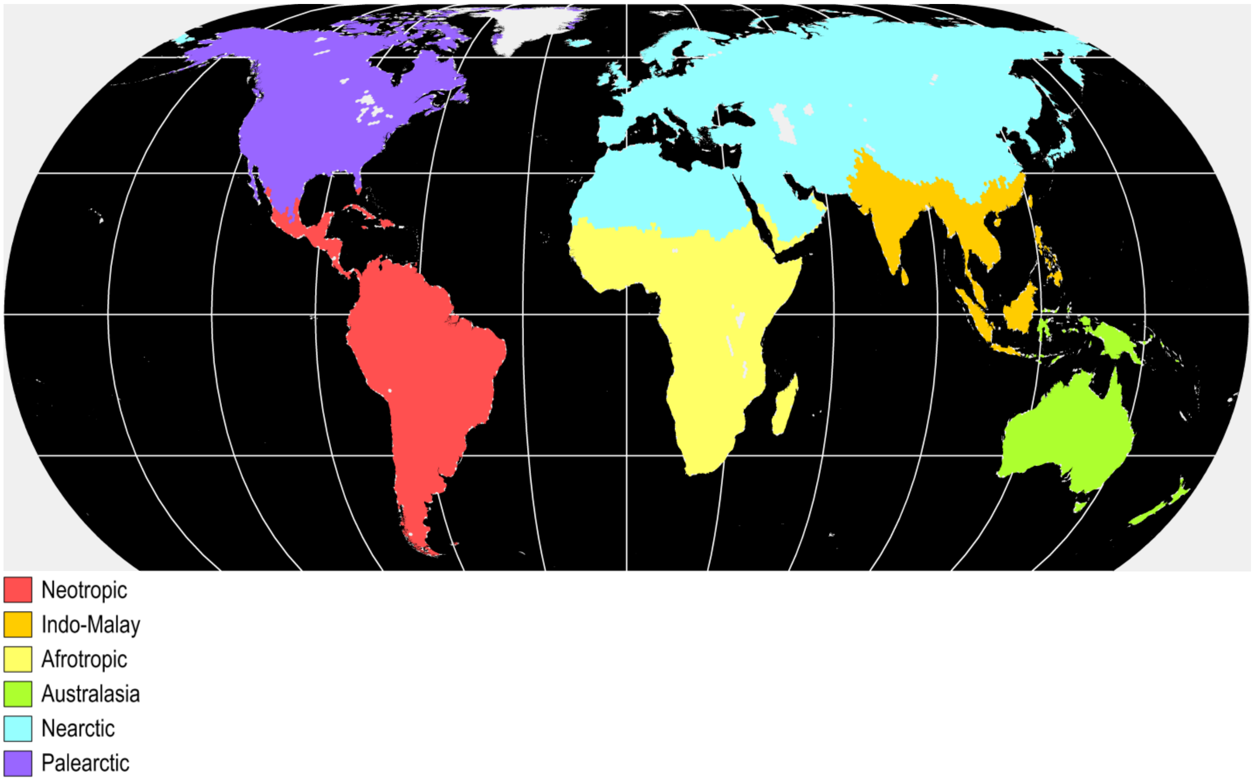

Figure 3: Biodiversity Hotspots (Mittermeier *et al.* 2005)

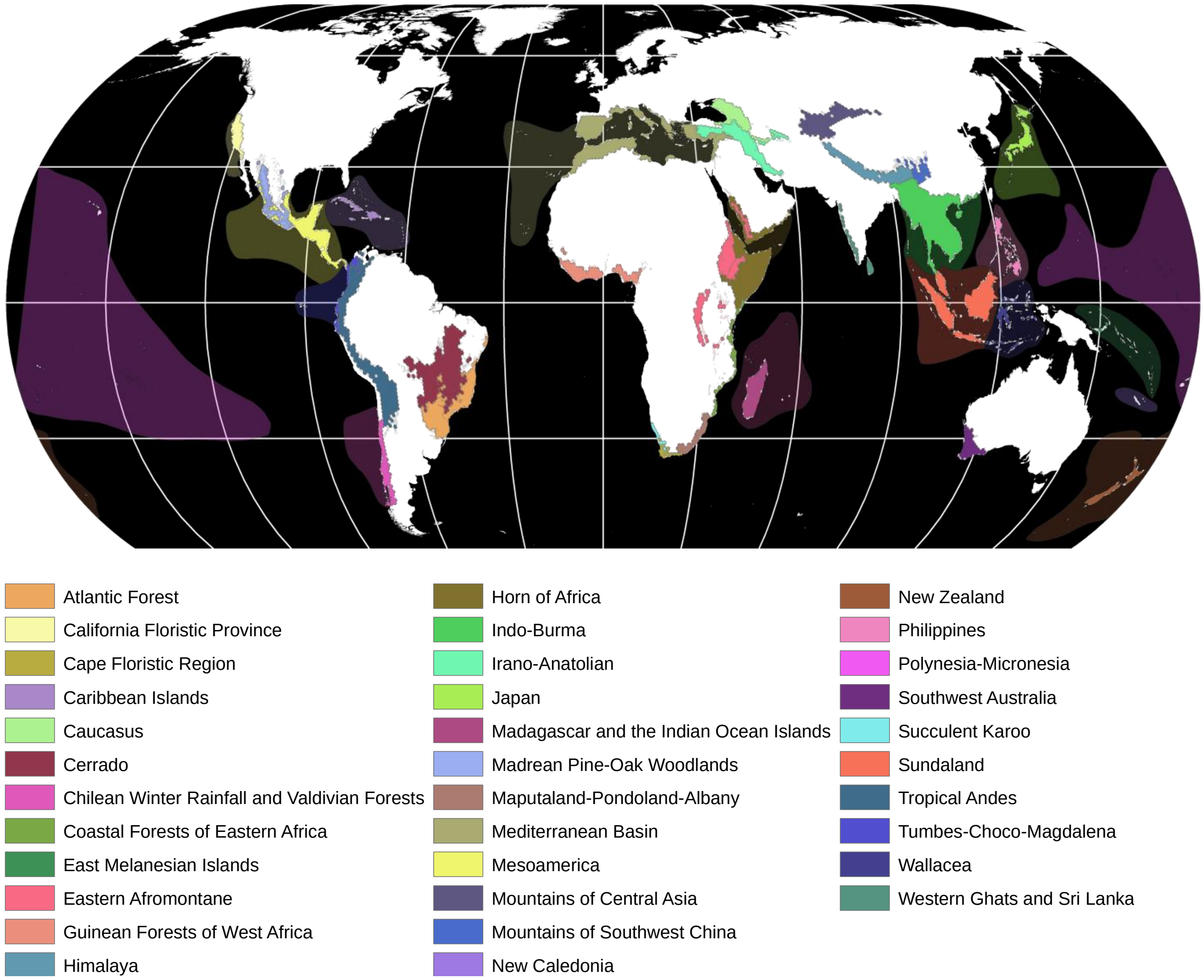

Figure 4: Anthromes (v2; Ellis *et al.* 2010)

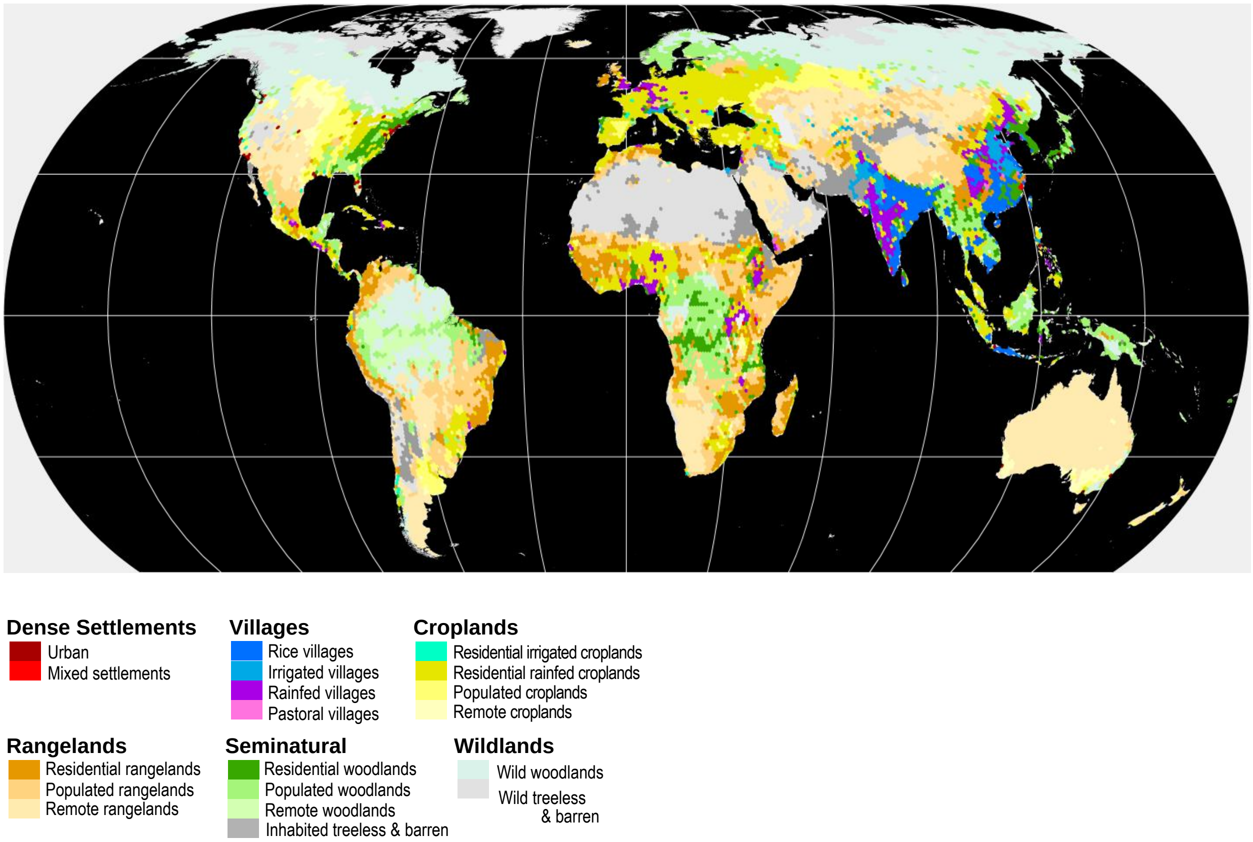

Figure 5: Crop Species Richness (*CS*; based on Monfreda *et al.* 2008; Appendix 1).

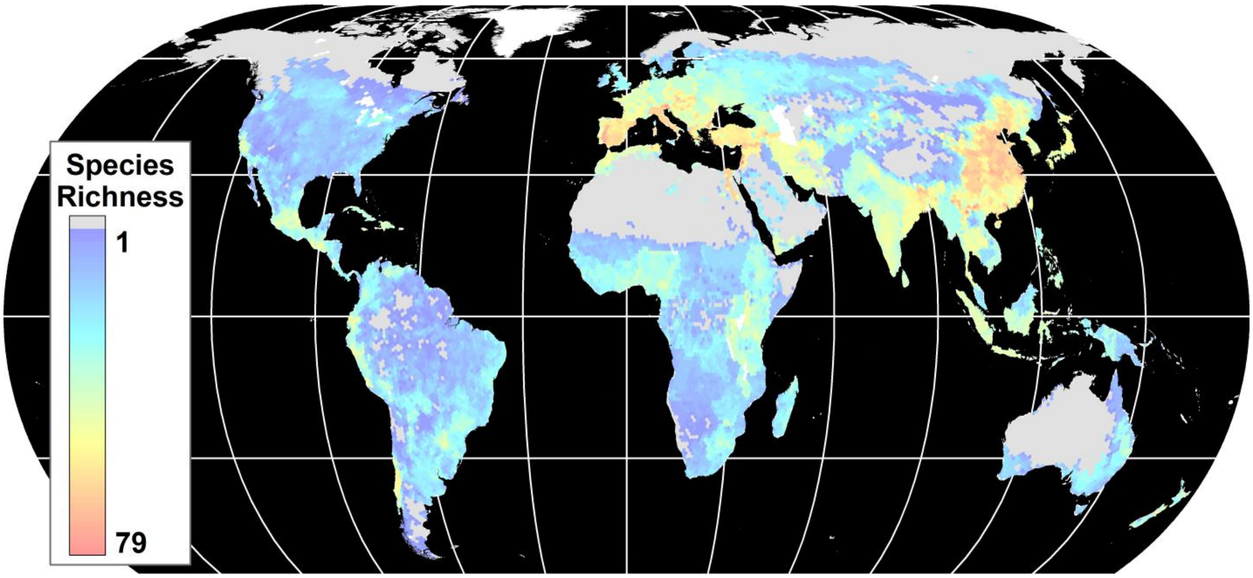

Figure 6: Ornamental Species Richness (*OS*; prepared as detailed in Appendix 1).

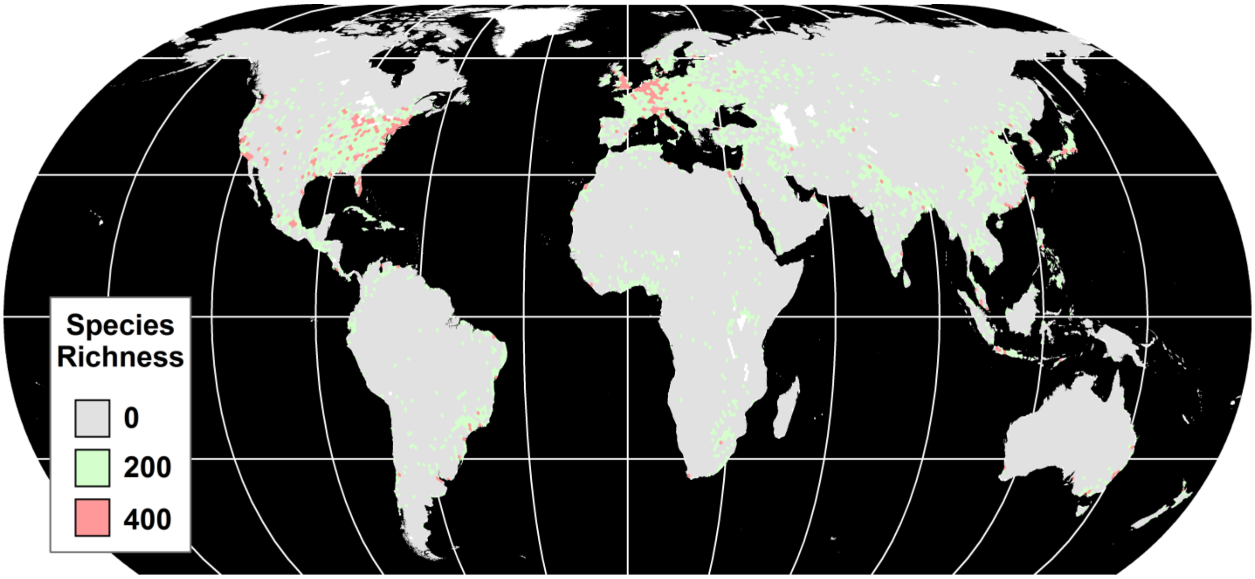

References Cited

Ellis EC, Klein Goldewijk K, Siebert S, Lightman D, Ramankutty N (2010) Anthropogenic transformation of the biomes, 1700 to 2000. *Global Ecology and Biogeography,* **19**, 589-606.

Mittermeier RA, Gil PR, Hoffman M *et al.* (2004) *Hotspots Revisited: Earth's Biologically Richest and Most Endangered Terrestrial Ecoregions*, Conservation International.

Monfreda C, Ramankutty N, Foley JA (2008) Farming the planet: 2. Geographic distribution of crop areas, yields, physiological types, and net primary production in the year 2000. *Global Biogeochemical Cycles,* **22**, GB1022.

Olson DM, Dinerstein E, Wikramanayake ED *et al.* (2001) Terrestrial ecoregions of the world: a new map of life on Earth. *BioScience,* **51**, 933-938.
